# Comparison enteral superoxide dismutase 1 IU and 5 IU from *Cucumis melo* L.C extract combined with *gliadin* as an antioxidant and anti-inflammatory in LPS-Induced sepsis model rats

**DOI:** 10.1016/j.heliyon.2022.e10236

**Published:** 2022-08-18

**Authors:** Cut Meliza Zainumi, Gontar Alamsyah Siregar, Dadik Wahyu Wijaya, Muhammad Ichwan

**Affiliations:** a Department of Anesthesiology and Intensive Care, Faculty of Medicine, Universitas Sumatera Utara, Medan, Indonesia; b Department of Internal Medicine, Faculty of Medicine, Universitas Sumatera Utara, Medan, Indonesia; c Department of Pharmacology and Therapeutic, Faculty of Medicine, Universitas Sumatera Utara, Medan, Indonesia

**Keywords:** *Cucumis melo* L.C. gliadin, SOD extract, Antioxidant, Sepsis model, MDA, TNF-α, Lactate

## Abstract

Sepsis is a major cause of death in intensive care units whose development is supported by an imbalance of oxidative stress and antioxidant. *Superoxide dismutase* (SOD) is a primer endogen antioxidant that prevents *reactive oxygen species* (ROS). Extensive studies on animals and humans have examined *Cucumis melo* L.C, a cantaloupe rich in SOD, and its combination with *gliadin*. The studies aimed to determine the effect of enteral administration of *Cucumis melo* L.C. gliadin (CME-gliadin) 28 days before inducing sepsis in rats. This experimental study aimed to compare four groups of male Wistar rats, including negative and positive control rats and those supplemented with SOD CME-gliadin 1 IU/day and SOD CME-gliadin 5 IU/day. All rats were given the same standard, except the supplementation for 28 days. Sepsis was induced by intraperitoneal injection of LPS 10 mg/kg. Enteral administration of SOD – gliadin extract of CME-gliadin for 28 days was used as antioxidant prophylaxis against oxidative stress due to sepsis. The results showed that enteral administration of CME-gliadin of 1 IU/day and 5 IU/day significantly increased SOD levels based on examination after 14 and 28 days. Also, it significantly decreased MDA (p < 0.001), TNF-α (p < 0.001), and lactate levels in rats induced by sepsis. However, the increase in lactate levels was above >1.64 mmol/l, indicating a high mortality rate. There was no significant difference in SOD, MDA, TNF-α, and Lactate levels between SOD 1 IU and SOD 5 IU. This descriptive data show that SOD 5 IU has a better result in MDA, TNF-α, and Lactate levels than SOD 1 IU.

## 1 Introduction

Sepsis is an excessive stimulus response from an organism where classic pathways are preoccupied with the Lipopolysaccharide (LPS) from Gram-negative bacteria. Some bacterial products, such as peptidoglycan and lipoteichoic acid from *Staphylococcus aureus* and other microorganisms trigger the systemic inflammatory response. The intensity of this inflammatory response is related to the interaction of immune cells (Rosario and Azevedo, 2008; Cecconi et al., 2018). During this response, some inflammatory mediators such as cytokine and chemokine increase circulation. The production of cytokines activates leukocytes and increases free radicals, such as reactive oxygen species (ROS) and nitrogen species (RNS) (Rocha, R. Herance et al., 2012). Furthermore, toll-like receptors (TLRs) initiate the activation of nuclear factor kappa B (NF-κB) and stimulate pro-inflammatory cytokines such as tumor necrosis factor-alpha (TNF-α), interleukin-1 (IL-1), and interleukin 6 (IL-6). This triggers the activation of the complement pathway, stimulating pro coagulation. Cytokine stimulates immune, endothelial, and epithelial cells to increase ROS, such as superoxide (O_2_), nitric oxide (NO), and peroxynitrite (ONOO^−^) (Kaymak et al., 2012).

Superoxide dismutase (SOD) is an endogenous antioxidant found in all organisms using oxygen as a life source. It catalyzes the conversion of superoxide into H_2_O, a reaction considered the primary antioxidant defense that prevents the occurrence of ROS. Another important enzyme is Glutathione peroxidase (GPx), which uses GSH as a co-factor inside cells. It contains selenium, essential in the removal of H_2_O_2_. The enzyme GPx converts hydrogen peroxide into water, the same role of catalase (CAT) domiciled in the perixome. Catalase becomes crucial when the hydrogen peroxide concentration increases because their reaction is faster than glutathione peroxidase (Rosario and Azevedo, 2008). Additionally, superoxide readily reacts with NO to form peroxynitrite (ONOO^−^), a strong oxidizing agent that stimulates DNA fragmentation, membrane damage, and lipid peroxidation (Abreu and Cabelli, 2010).

Kumar et al. (2018) found that SOD levels are reduced significantly in sepsis than in control patients. A sepsis study in children also found that superoxide dismutase was lower than controls (Molina et al., 2017).

Regulating antioxidants such as SOD, glutathione peroxidase, and catalase balances the mitochondria. Therefore, an increase in endogenous antioxidants prevents mitochondrial damage. Administering intravenous mimetic SOD also prevents hypotension and pro-inflammatory cytokines and reduces mortality in rat models of sepsis. Moreover, up-regulation of SOD by insulin induction protects mitochondria from oxidative stress in acute renal failure performed in septic mice (Salvemini et al., 2002; Macarthur et al., 2003; Borgstahl and Oberley-Deegan, 2018; Nagar et al., 2018).

The recommended antioxidants are vitamins E and C and selenium, zinc, and copper minerals. However, the administration route, frequency, and duration have not been standardized (Manzanares et al., 2012; McClave et al., 2016; Singer, M. et al., 2016; Carr, 2019; Li et al., 2019; Weiss, Balamuth, et al., 2020; Weiss, Co-vice et al., 2020). This implies room for studies on antioxidants to optimize enteral nutrition in septic patients.

Oral antioxidant supplements are popular in western countries, especially in animals and humans. One reason for the failure of these supplements may be due to reduced bioavailability, or their effects are not long-term. An example is SOD because its large molecular weight cannot penetrate cell membranes. Since 2000, melon extracts rich in SOD have been developed into dietary supplements. However, the pH and proteolytic activity of the gastrointestinal tract make SOD inactive and ineffective (Intes et al., 2012; Romao, 2015).

*Cucumis melo* Linnaeus Cucurbitaceae (*Cucumis melo* L.C.) is a melon fruit that contains 7 times more SOD than regular melon. Glisodin® is *Cucumis melo* L.C. extract rich in SOD coated by a wheat matrix polymer layer gliadin. Studies have found that gliadin carries SOD orally and increases adhesion enzymes into the gastrointestinal epithelium, making it easily absorbed in the small intestine (Vouldoukis, Krauss, et al., 2004). This combination of *Cucumis melo* L.C. gliadin has been widely studied in animals and humans, such as diabetes, reperfusion injury, fibrosarcoma, cognitive, atherosclerosis, and antiaging (Goldberg and Crysler, 2014; Romao, 2015).

This study aimed to determine the effect of enteral administration of *Cucumis melo* L.C. gliadin (CME-gliadin) to compare four groups of male Wistar rats, including negative and positive control rats and those supplemented with SOD CME-gliadin 1 IU/day and SOD CME-gliadin 5 IU/day as an antioxidant and anti-inflammatory in LPS-induced sepsis model rats.

## 2 Material and methods

### 2.1 SOD enteral

This study used Glisodin® as a SOD enzyme extracted from *Cucumis melo Linnaeus Cucurbitaceae* (*Cucumis melo* L.C.), *extract cell line* 95LS444 USA *Patent* 5,747,043 combined with wheat *gliadin* (*Triticum vulgare*) *biopolymer* to slow release of SOD. Glisodin® is a water-dispersible SOD containing 1 IU/mg of final dry powder of active SOD. Each capsule of Glisodin® contains 250 IU SOD extracted from *Cucumis melo* L.C. The 250 mg dry powder was dissolved in water, centrifuged into homogenous, and given to the rats by force-feeding.

### 2.2 Assessments of antioxidant and anti-inflammatory parameters

ELISA was used to measure SOD, MDA, TNF-α, and Lactate blood serum. SOD levels were measured in nmol/L using an ELISA SOD kit (Bioassay Technology Laboratory, Shanghai, China). Similarly, MDA levels were measured in nmol/L using ELISA *thiobarbituric acid reactive substance* (TBARS) kit (Bioassay Technology Laboratory, Shanghai, China). TNF-α levels were measured in ng/L using an ELISA TNF-α kit (Bioassay Technology Laboratory, Shanghai, China). Lactate levels were measured in mmol/L using an ELISA kit (MyBioSource, USA). The sample was mixed with lactate assay buffer and measured by calorimetric using absorbent 570 nm and the fluorometric using intensity 535–587 nm.

### 2.3 Animal model and induction to sepsis

This study obtained and adapted 32 male white Wistar rats for one week at the Testing Service Laboratory – Institute of Bioscience, Universitas Brawijaya Malang. All rats were given the same food and drink, except those given Glisodin® supplementations and cycles every 12 h of light and dark at 24 °C. The experimental protocol was performed in accordance with the ARRIVE (Animal Research: Reporting in Vivo Experiments) guidelines version 2.0. and already approved by the Health Research Ethics Committee of Universitas Sumatera Utara according to the Nuremberg Code (no: 554/KEP/USU/2020). Subsequently, sepsis was induced to animals by injecting LPS *Escherichia coli* serotype 0111:B4 obtained from Sigma-Aldrich (Singapore) 10 mg/kg intraperitoneal.

### 2.4 Experimental protocol

This experimental study used the Randomized Control Trial to compare the control group of rats with the group given *Cucumis melo* L.C gliadin extract for 28 days in the septic rat model. The animal models were maintained and induced with sepsis at the Testing Service Laboratory – Institute of Biosciences, Universitas Brawijaya Malang. The ELISA examination and analysis of the results were conducted at the Testing Service Laboratory – Institute of Biosciences, Universitas Brawijaya Malang.

The rats were weighed before the study, followed by random sampling and putting them into cages of four groups, each with eight rats. Group A rats were given food and drink as a control, while group B was given food, drink, and LPS after 28 days. In contrast, group C rats were given Glisodin® at a dose of 1 IU/day for 28 days, while group D was given Glisodin® at a dose of 5 IU/day for 28 days.

Blood samples were periodically collected before treatment, day-7, day-14, day-28, and after LPS injection. The samples were collected from retroorbital venous plexus, except after injection of LPS, where the animals were decapitated after anesthesia, and the blood was rapidly collected by direct heart puncture. Plasma samples were separated from blood cells by centrifugation at 3000 g for 10 min and stored frozen for use in the ELISA analysis.

### 2.5 Statistical analysis

Data were expressed as mean ± standard error of the mean (S.E.M.). One-way analysis of variance (ANOVA) was used to analyze the differences between the groups, followed by Tukey’s multiple comparisons using Graph Pad Prism 9.3.1 Software. A p < 0.05 was considered statistically significant for mean differences.

## 3 Results

### 3.1 Effect of CME-gliadin on SOD level

A randomized controlled trial was conducted on experimental rats to compare the control group with the group given *Cucumis melo* L.C gliadin extract for 28 days. The rats were adult male Wistar rats, aged 3–4 months, with a bodyweight of 146–327 g. White Wistar rats were obtained and adapted at the Testing Service Laboratory – Institute of Biosciences, Universitas Brawijaya Malang.

**Figure 1:**
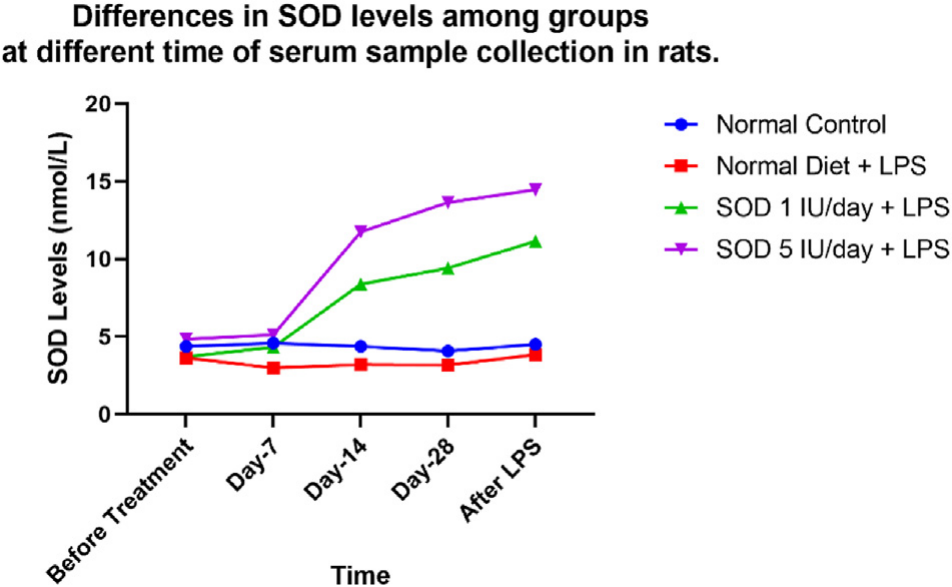
Differences in SOD levels among groups at different times of serum sample collection in rats. Group A normal control rats with a normal diet, Group B negative control rats with a normal diet with LPS induced, Group C supplemented SOD 1 IU/day diet with LPS induced, Group D supplemented SOD 5 IU/day with LPS induced.

Table 1 shows the results of examining SOD levels in each group. There was no significant difference in SOD levels between groups before treatment and SOD examination on day-7 (p = 0.898 and p = 0.626). However, significant differences were found on days 14, 28, and after LPS. The levels of SOD from the highest to the lowest were groups D, C, B, and A. Figure 1 shows changes in SOD levels from before treatment to after LPS. SOD levels in groups C and D increased on days 14, 28, and after LPS.

**Table 1:** Differences in SOD levels among groups at a different time of serum sample collection in rats.

Variable	Mean (nmol/L)	SD (±)	Minimum-Maximum	P-value
SOD before treatment				
Group A	4.38	1.941	0.10–5.89	
Group B	3.90	2.182	0.06–6.23	0.898^a^
Group C	4.24	2.360	0.25–8.13	
Group D	4.83	0.568	4.02–5.95	
SOD day-7				
Group A	4.59	0.735	3.14–5.16	
Group B	3.98	1.99	1.23–6.45	0.626^a^
Group C	4.93	1.597	3.25–8.23	
Group D	5.12	0.780	4.02–6.18	
SOD day-14				
Group A	4.37	0.624	3.44–5.33	
Group B	4.27	1.717	2.12–6.19	<0.001^a^
Group C	9.57	2.626	5.21–13.92	
Group D	13.42	13.428	10.68–17.98	
SOD day-28				
Group A	4.07	0.934	2.48–5.23	
Group B	4.24	1.621	2.47–6.91	<0.001^b^
Group C	10.75	2.539	6.34–14.12	
Group D	15.59	1.922	12.85–18.92	
SOD after LPS				
Group A	4.51	0.929	2.53–5.43	
Group B	5.12	0.671	4.45–6.34	<0.001^b^
Group C	12.73	3.877	7.12–19.48	
Group D	16.52	1.629	14.21–19.21	

Group A normal control rats with a normal diet, Group B negative control rats with a normal diet with LPS induced, Group C supplemented SOD 1 IU/day diet with LPS induced, Group D supplemented SOD 5 IU/day with LPS induced.

a Kruskal-Wallis test ANOVA test with significance p < 0,5.

There was a significant difference between overall SOD, with a p-value < 0.0001 using one-way ANOVA. Statistic and post-hoc tests showed no significant difference between SOD 1 IU and 5 IU, with a p-value of 0.2134.

### 3.2 Effect of CME-gliadin on MDA level

Analysis of differences in MDA after LPS levels between groups was conducted using the Kruskal Wallis test. The analysis results in Table 2 show a significant difference with a p = 0.001 in the MDA levels in each group. Group D had the lowest MDA levels compared to groups C and B. MDA levels in group D were almost the same as group A, which showed levels of 2.77. Group B, C, and D showed 5.64, 2.86, and 2.63 levels, respectively.

**Table 2:** Effects of SOD CME-gliadin supplement on MDA serum levels in rats.

Variable	Mean (nmol/mL)	Minimum-Maximum	P-value
MDA after LPS			
Group A	3.16	0.03–4.00	
Group B	19.83	13.84–33.34	0.001^a^
Group C	3.92	3.59–4.24	
Group D	3.24	0.04–4.40	

Group A normal control rats with a normal diet, Group B negative control rats with a normal diet with LPS induced, Group C supplemented SOD 1 IU/day diet with LPS induced, Group D supplemented SOD 5 IU/day with LPS induced.

a Kruskal-Wallis test with significance p < 0,5.

There was a significant difference between overall MDA with all groups with a p-value < 0.0001 using one-way ANOVA. Statistic and post-hoc tests showed no significant difference between SOD 1 IU and 5 IU, with a p-value of 0.9956.

**Figure 2:**
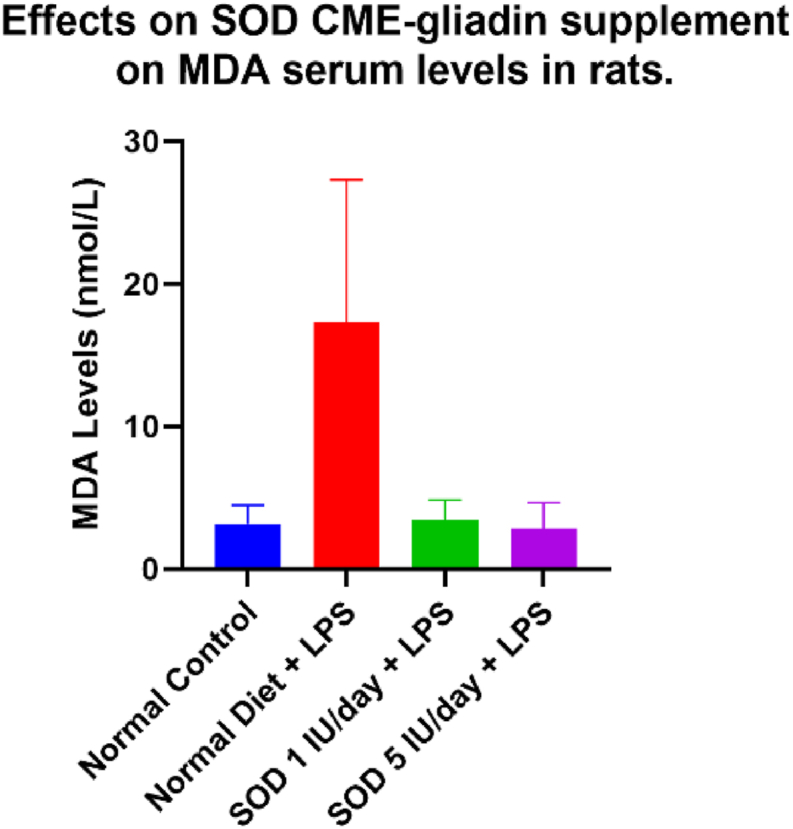
*Effects of SOD CME-gliadin supplement on* MDA *serum* level*s in rats.* Group A normal control rats with a normal diet, Group B negative control rats with a normal diet with LPS induced, Group C supplemented SOD 1 IU/day diet with LPS induced, Group D supplemented SOD 5 IU/day with LPS induced.

In this study, we found that MDA in rats that treated by SOD either 1 IU and 5 IU had a lower MDA, as shown in Figure 2, even there’s no difference in different dose based on statistics measurement, we could still see that in Group D (5 IU) the mean of MDA are lower than in Group C (1 IU).

### 3.3 Effect of CME-gliadin on TNF-α level

Table 3 shows the analysis results of differences in TNF-α after LPS levels using the ANOVA test analysis. The results show significant differences in TNF-α levels in each group. Group D had the lowest TNF-α levels compared to group A.

**Table 3:** Effects of SOD CME-gliadin supplement on TNF-α serum levels in rats.

Variable	Mean (ng/L)	SD (±)	Minimum-Maximum	P-value
TNF-α after LPS				
Group A	2.77	0.454	1.97–3.44	
Group B	5.64	1.835	2.45–7.66	<0.001^b^
Group C	2.86	0.401	2.20–3.35	
Group D	2.63	0.694	1.72–4.00	

Group A normal control rats with a normal diet, Group B negative control rats with a normal diet with LPS induced, Group C supplemented SOD 1 IU/day diet with LPS induced, Group D supplemented SOD 5 IU/day with LPS induced.

b Anova test with significance p < 0,5.

There was a significant difference between overall TNF-α with all groups, with a p-value < 0.0067 using one-way ANOVA. Statistic and post-hoc tests showed no significant difference between SOD 1 IU and 5 IU, with a p-value of 0.9938.

**Figure 3:**
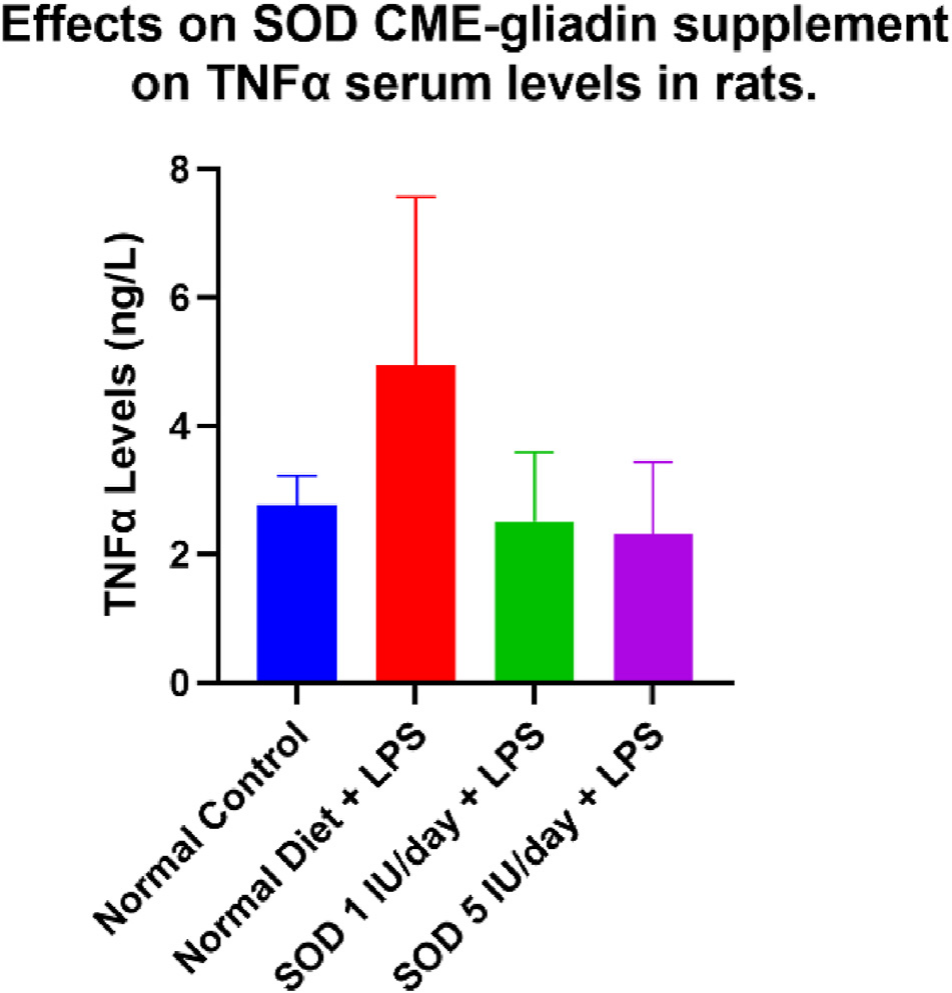
Effects of SOD CME-gliadin supplement on TNF-α serum levels in rats. Group A normal control rats with a normal diet, Group B negative control rats with a normal diet with LPS induced, Group C supplemented SOD 1 IU/day diet with LPS induced, Group D supplemented SOD 5 IU/day with LPS induced.

In this study, the result of TNF-α we found caused by inflammatory response and oxidative stress from production of cytokine pro-inflammation. SOD that was given shows us anti-inflammatory effect, even there’s no difference in different dose, 1 IU and 5 IU. We could still see in Figure 3 that in Group D (5 IU) the mean of TNF- α are lower than in Group C (1 IU).

### 3.4 Effect of CME-gliadin on lactate level

There was a significant difference in lactate levels after LPS measurement (p < 0.001). Group A had the lowest lactate levels among the groups that received the intervention, with a mean of 1.18, as shown in Table 4.

**Table 4:** Effects of SOD CME-gliadin supplement on Lactate serum levels in rats.

Variable	Mean (mmol/L)	SD (±)	Minimum-Maximum	P
Lactate after LPS				
Group A	1.18	0.299	0.90–1.70	
Group B	3.88	0.598	3.10–4.70	<0.001^a^
Group C	3.47	0.256	3.10–3.80	
Group D	3.15	0.454	2.30–3.60	

Group A normal control rats with a normal diet, Group B negative control rats with a normal diet with LPS induced, Group C supplemented SOD 1 IU/day diet with LPS induced, Group D supplemented SOD 5 IU/day with LPS induced.

a Kruskal-Wallis test with significancy p < 0,5.

There was a significant difference between overall Lactate with all groups, with a p-value < 0.0033 using one-way ANOVA. Statistic and post-hoc tests showed no significant difference between SOD 1 IU and 5 IU, with a p-value of 0.9631.

**Figure 4:**
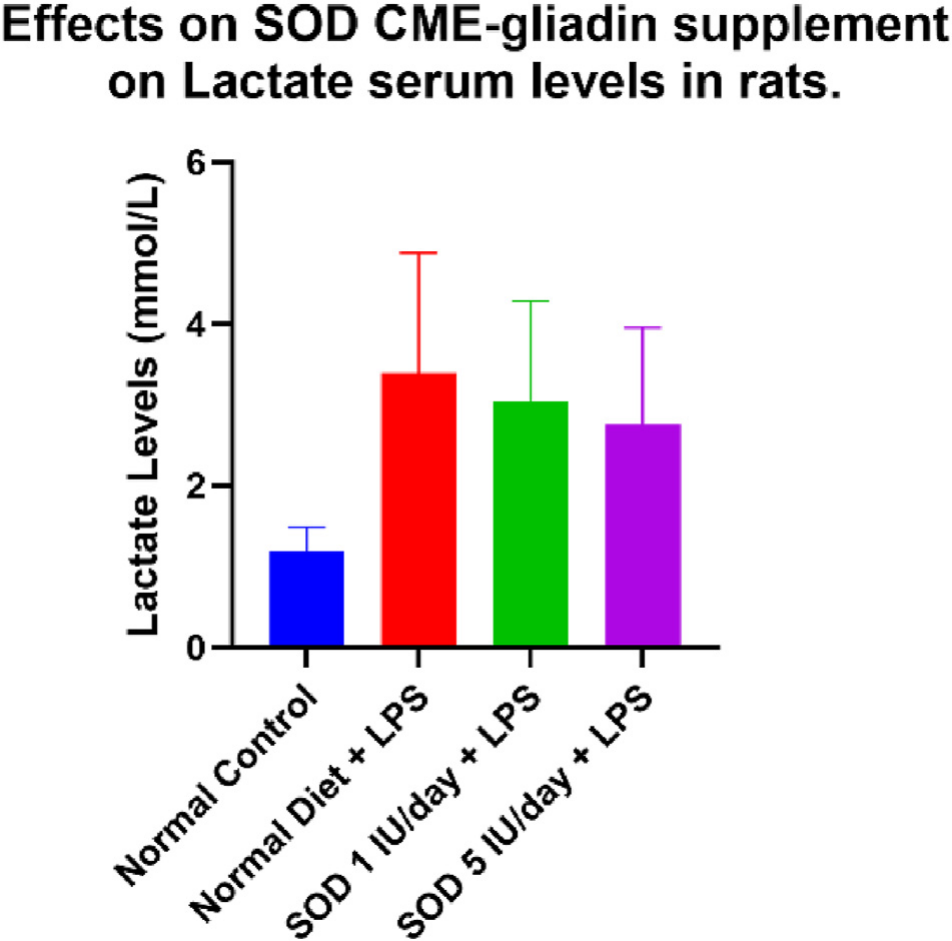
Effects of SOD CME-gliadin supplement on Lactate serum levels in rats. Group A normal control rats with a normal diet, Group B negative control rats with a normal diet with LPS induced, Group C supplemented SOD 1 IU/day diet with LPS induced, Group D supplemented SOD 5 IU/day with LPS induced.

In this study, the lactate serum level remains high in rats with supplemented SOD either 1 IU and 5 IU, as shown in Figure 4, this could happen because the rats didn’t receive any sepsis treatment and only given SOD as the treatment. Lactate serum level would be decreased if the rats received the main treatment for the main problem such as resuscitation, medication of sepsis, etc. This study only give SOD to know the effect of SOD as an adjunctive therapy for sepsis.

### 3.5 Comparison effect of CME-gliadin on SOD 1 IU and SOD 5 IU

The one-way ANOVA test results necessitated determining the difference between SOD 1 IU and 5 IU using a post-hoc test. Tukey’s multiple comparisons showed no significant difference in SOD, with a p-value of 0.2134, MDA with a p-value of 0.9956, TNF-α with a p-value of 0.9938, and Lactate with a p-value of 0.9631.

The descriptive result showed that SOD was higher in group 5 IU than in 1 IU. There was a lower MDA, TNF-α, and Lactate in group 5 IU than 1 IU, with no significant difference.

## 4 Discussion

Sepsis pathogenesis is a complex mechanism involving interaction between microbial and host responses. Dysregulations from immune host response in sepsis occur from uncontrolled release of mediator, causing organ failure (Rocha, R Herance et al., 2012). The immune response is activated by interacting with microbial antigens called *pathogen-associated molecular patterns* (PAMPs). The activation is caused by *Toll-like receptors* (TLRs) and *nuclear factor kappa B* (NF-κB), transcription of pro-inflammatory cytokines IL-1α, IL-1β, TNF-α, IG-CSF, GM-CSF, adhesion molecules ICAM-I, VCAM-1, coagulation factors, prooxidant enzymes iNOS, COX-2, LOX, antioxidants SOD, GPx, and cel T. Furthermore, several products deliver positive feedback to NF-κB, enhancing inflammatory response. Signal transfer from ROS and RNS activates leucocytes, resulting in superoxide called ROS. The superoxide interacts with NO to form peroxynitrite (ONOO^−^), a potent oxydator that fragments the DNA, damages cell membranes and causes lipid peroxidation (Murphy, 2009; Abreu and Cabelli, 2010; Bhan et al., 2016; Jones et al., 2016; Acuña-Castroviejo et al., 2017).

Superoxide is produced from the electron transport chain in mitochondria and irreversibly inhibits electron transport and ATP synthesis with its peroxynitrite form. The main antioxidant that regulates superoxide to prevent cell damage is superoxide dismutase and glutathione cycle (Duran-Bedolla et al., 2014; Acuña-Castroviejo et al., 2017; Nagar et al., 2018).

This study aimed to determine the benefits of giving *Cucumis melo* L.C Gliadin to SOD levels as antioxidant prophylaxis in adult Wistar rats as sepsis models. The samples tested comprised 32 mice divided into group A as a control, B as rats given LPS injection on day 28 as a sepsis model, and C and D with a prophylactic intervention with Glisodin® with a dose of 1 IU/day and 5 IU/day.

The results showed that SOD levels increased significantly on days 14 and 28. The time required for an increase in SOD levels was in line with Vouldoukis et al. (Vouldoukis, Krauss, et al., 2004), which administered SOD-gliadin extract for 28 days. SOD levels increased on day 14 and reached a maximum effect after 28 days. Administration of SOD through plant extracts containing antioxidants is ineffective due to the denaturation of antioxidants, especially when given orally. This could be overcome by wrapping the SOD using lipids and proteins. Also, wheat gliadin contained in Glisodin® s useful as a drug modality to maintain the bioavailability of SOD, even when taken orally (Carillon et al., 2013).

Gliadin protects SOD from gradation in the stomach due to its bio-adhesion properties. It increases the stickiness of SOD to the small intestinal epithelium, prolonging the association of SOD with the gastrointestinal tract (Intes et al., 2012). Furthermore, gliadin biopolymers trap and delay the release of the active ingredient during digestion. This is due to its bioadhesive properties, which enhance the delivery of the active ingredient in the intestinal mucosa (Carillon et al., 2013).

Sepsis induction causes ROS damage when the oxidant produced exceeds the capacity of endogenous SOD. Oral supplementation and direct ROS detoxification also increase endogenous antioxidant defenses. Previous studies showed that *Cucumis melo* L.C. gliadin extract is an antioxidant nutrient-rich extract. It has high SOD of 100 IU/NBT per mg dry extract on average, catalase (10 IU/mg), natural antioxidants, and glutathione peroxidase. Antioxidants reduce oxidative stress, while heterologous SODs or antigens may have immunoregulatory properties (Vouldoukis, Krauss, et al., 2004).

The crude *Cucumis melo* L.C. gliadin extract with SOD activity inhibited the production of superoxide anion in a dose-dependent manner by IgG1IC-stimulated macrophages. The maximal inhibitory effect was achieved at 100 ng/ml *Cucumis melo* L.C. gliadin equivalent to 10 IU/NBT SOD activity. However, there was a significant difference in the inhibitory effect with heat-inactivated *Cucumis melo* L.C gliadin because HI-*Cucumis melo* L.C gliadin lacks SOD activity. This indicated that SOD activity was essential for reducing oxidative stress, though other antioxidant products reduced superoxide anion production by IgG1IC-treated macrophages (Vouldoukis, Lacan, et al., 2004).

Tumor Necrosis Factor-Alpha (TNF-α) is a pro-inflammatory cytokine expressed by septic mouse model tissue. This study showed that TNF-α levels were significantly different in each group. Specifically, groups A, B, C, and D had 2.77 ng/L, 5.64 ng/L, 2.86 ng/L, and 2.63 ng/L, respectively. A p-value of <0.001 showed a significant difference in TNF-α after LPS levels between groups.

Previous studies found that oxidative metabolism is timely related to macrophages' pro and anti-inflammatory capacity. The studies evaluated the effects of various extracts of *Cucumis melo* L.C. gliadin-induced IgG1IC on TNF-α and IL-10 production by macrophages. Stimulation of IFN-gamma-activated macrophages by IgG1IC induces the simultaneous production of TNF-α and IL-10. The observed inverse correlation between TNF-α and IL-10 concentrations indicates a physiological balance between the production processes of the two cytokines. After supplementing *Cucumis melo* L.C. gliadin extract, IgG1IC-induced TNF-α production in IFN-gamma-activated macrophages was significantly reduced (P < 0.01), while IL-10 production was significantly increased (P < 0.01). Therefore, the effect of SOD on *Cucumis melo* extract may decrease pro-inflammatory and increase anti-inflammatory cytokine levels (Vouldoukis, Lacan, et al., 2004).

LPS injection causes an inflammatory response and oxidative stress by producing pro-inflammatory cytokines such as TNF-α and IL-6. Administering SOD inhibits the inflammatory process and oxidative stress in acute inflammation caused by LPS (Porfire, 2014). Also, Glisodin® administration significantly reduces TNF-α levels in endometriosis rats (Trisetiyono et al., 2019).

Lipid peroxidase occurs because oxidants such as free radicals or non-radicals attack lipids containing double-bonded carbon, especially polyunsaturated fatty acids (PUFAs). The reaction is caused by malondialdehyde (MDA), a biomarker for poor prognosis in sepsis (Ansarin et al., 2015; Lorente et al., 2015). This study found a significant relationship where MDA levels were lower in the group given *Cucumis melo* L.C. gliadin extract. Group A mice not induced with LPS sepsis had relatively comparable MDA levels than group D induced by sepsis and treated with *Cucumis melo* L.C. gliadin extract. Moreover, there were significant differences in MDA levels in each group. These results explain the benefits of SOD on lipoperoxidation resulting in MDA. Similar results were found by Trisetiyono et al. (2019) after inducing rats with endometriosis and *Cucumis melo* L.C. gliadin extract.

Administering a dose of 5 IU significantly reduced MDA and TNF-α levels. Another study in mice given CME-gliadin exposed to second-hand smoke for 28 days also showed lower serum MDA levels. The mice were exposed to cigarette smoke and given doses of SOD-gliadin 2.25 IU, 4.5 IU, and 9 IU, respectively. This study found that administering 9 IU of SOD insignificantly reduced MDA levels. In contrast, a dose of 2.25 IU significantly reduced MDA levels compared to the negative control group without SOD administration. Therefore, administering CME extract increases antioxidant capacity and reduces oxidative stress, characterized by a decrease in MDA levels (Suryadinata et al., 2017).

The results showed a significant difference in lactate levels after LPS measurement, where the rats were induced with sepsis. This implies a relationship between LPS administration to lactate levels in sepsis model rats. Lactate is a biomarker of hypoperfusion that helps in resuscitating septic patients. Recent studies have considered assessing whether baseline lactate is a risk stratification biomarker or an additional manifestation of organ dysfunction (Tredway et al., 2011). Similar results were found by Zhai et al. after inducing rats with sepsis by the CLP method. The study also investigated and established a lactate cut-off value of >1.64 mmol/L predicting sepsis in rats. According to Zhai et al. (2018), rats with lactate values >1.64 had a 100% mortality rate. The results support this study, which found that all rats given LPS had lactate levels >1.64, meaning they had sepsis. Lactate is a normal product in glycolysis, which increases in sepsis, while pyruvate dehydrogenase decrease when lactating levels, except in tissue hypoxia (Tapia et al., 2015). In hypoxic situations, cells produce ATP through glycolysis using pyruvate to lactate rather than acetyl-CoA. This occurs even under aerobic glycolysis or the Warburg effect (Pearce and Pearce, 2013). The result is increased production and a significant decrease in lactate clearance in sepsis (Hernandez et al., 2019). However, this study did not perform resuscitation or medication on sepsis rats.

Lactate as a diagnostic marker and as a marker of disease progression has a strong association with disease severity and patient outcome. Blood lactate is a marker of abnormal microcirculation, reflective of tissue hypoperfusion, and cellular hypoxia. An increased lactate level is a sign of cellular dysfunction in sepsis, such as insufficient tissue oxygen delivery, impaired aerobic respiration, and accelerated aerobic glycolysis. In septic animals, persistent increased lactate shows a severe infection and inflammation, worse hemodynamic abnormality, and more serious organ dysfunction. Persistent elevation of serum-lactate would need adequate volume substitution and need for vasopressor therapy to maintain a mean arterial pressure >65 mmHg (Zhai et al., 2018; Jarczak et al., 2021). Based on that we know that lactate would still be elevated if the rats didn’t receive any resuscitation, hemodynamic management, and medication treatment for sepsis.

Glisodin® extract has been administered to animals and humans in various studies with no adverse side effects. Studies have been conducted on cancer, cardiovascular, degenerative, and infectious diseases (Webb et al., 2008; Nakajima et al., 2009; Carillon et al., 2013; Porfire, 2014; Romao, 2015; Subandrate et al., 2015; Ahasan, 2019). These diseases are related to ROS production, a condition improved by manipulating antioxidant levels.

Oxidative stress is triggered by hyperbaric oxygen (HBO) conditions. HBO therapy treats various diseases with possible side effects, especially DNA damage. The results showed a significant reduction in DNA strand breaks in the SOD-gliadin-treated group compared to the placebo group. Additionally, treated patients showed decreased plasma marker concentrations for oxidative stress (Muth, 2004). The anti-inflammatory effect of Glisodin® prevents the development of many chronic inflammation-mediated diseases. According to Okada et al. administering the gliadin-SOD complex could prevent the development of cancer (Romao, 2015).

Cloarec et al. recruited 76 patients considered at risk for cardiovascular disease but free of clinical signs. In the study, 17 subjects were given Glisodin® 500 IU for two years. The result showed that supplementation causes a 34% decrease in malondialdehyde (MDA) levels, atherosclerosis, and cardiovascular disease pathogenesis. Moreover, the effects of Glisodin® were seen on stress-induced lipid peroxidation and impaired spatial memory in mice. Glisodin® protects against lipid peroxidation in nerve cells and prevents the decline in rat spatial memory. Furthermore, it has a neuroprotector effect by secreting nerve growth factor (NGF) and insulin-like growth factor (IGF-1). Intake of Glisodin® containing oral bioactive SOD may significantly improve quality of life. Most studies on SOD-gliadin formulations show that supplementation provides additional effects than curative properties (Romao, 2015).

This study also found a significant difference in TNF-α and MDA between the test groups. However, the lactate and mortality values were still high, and the rats were declared septic, implying the need for standard sepsis. Administering SOD-gliadin to increase endogenous SOD could reduce the pro-inflammatory effect on sepsis. Therefore, further studies should use similar methods to prove the antioxidant effect of SOD-gliadin and standard therapy on sepsis.

No study has examined the difference in the effect of SOD 1 IU and SOD 5 IU doses of SOD. This study found no significant difference between SOD 1 IU and SOD 5 IU. Only SOD has a significant result with a good effect in septic rats, but the difference in doses of 1 IU and 5 IU is insignificant.

## 5 Conclusion

Enteral administration of SOD – gliadin extract of *Cucumis melo* L.C. gliadin for 28 days is useful as antioxidant prophylaxis against oxidative stress due to sepsis. Administering Glisodin® at 1 IU/day and 5 IU/day significantly increased SOD levels based on examination on days 14 and 28. Furthermore, enteral administration of SOD – gliadin extract of *Cucumis melo* L.C. gliadin decreased MDA levels significantly in sepsis-induced mice, with p < 0.001. Administering SOD – gliadin extract of *Cucumis melo* L.C. gliadin significantly reduced TNF-α levels in sepsis-induced rats, with p < 0.001.

Enteral administration of SOD – gliadin extract of *Cucumis melo* L.C. gliadin significantly reduced lactate levels in sepsis-induced rats. However, the increase was still above >1.64 mmol/l, implying a high mortality rate. There was no difference between SOD 1 IU and 5 IU, though descriptive data showed that SOD 5 IU has a better result in MDA, TNF-α, and Lactate levels than SOD 1 IU. Further studies should be conducted with a longer period or larger dose with shorter SOD – gliadin intake time.

## Declarations

### Author contribution statement

Cut Meliza Zainumi: Conceived and designed the experiments; Performed the experiments; Wrote the paper.

Gontar Alamsyah Siregar: Conceived and designed the experiments; Wrote the paper.

Dadik Wahyu Wijaya and Muhammad Ichwan: Analyzed and interpreted the data; Contributed reagents, materials, analysis tools or data.

### Funding statement

This research did not receive any specific grant from funding agencies in the public, commercial, or not-for-profit sectors.

### Data availability statement

Data will be made available on request.

### Declaration of interests statement

The authors declare no conflict of interest.

### Additional information

No additional information is available for this paper.
